# The Pharmacology of the Newer Materia Medica

**Published:** 1890-10

**Authors:** 


The Pharmacology of the Newer Materia Medica. Embracing the Botany, Chemistry, Pharmacy and Therapeutics of New Remedies. Being the results of the Collective Investigation of New Remedies, under the "Working Bulletin" System, Properly Arranged, Classified, Indexed and Placed at the Disposal of the Medical Profession. Issued in monthly parts. Subscription price, $2.00, in advance; single copies, twenty-five cents each. George S. Davis, Detroit, Mich. 1889.

This work is divided into subjects, each subject representing one of the new drugs. Under the heading of each drug is found:

1. The botany of the drug, embracing names, synonyms, natural order, origin, history, commerce, production, description, microscopical structure, etc.

2. The chemistry of the drug, composition, analysis, etc.

3. The pharmacy of the drug, adulterations and substitutes, pharmaceutical preparations, incompatibles, etc.

4. The therapeutics of the drug.

1. Reports of experiments made upon animals to determine the physiological action of the drug.

2. Clinical reports, pro and con, published in medical periodicals, etc., arranged with reference to the diseases in which the drug has been tested, or with reference to the nature of its action upon the human system.

3. Resumé by a competent physician, giving the indications, antagonists, synergists, physiological action, toxicology and antidotes, dosage, etc., of the drug, as established by the reports of clinical and physiological investigators, above mentioned.

Five parts have thus far appeared, and the publisher is deserving of much credit and praise for the great pains taken to give the medical profession such reliable information in so compact and usable a form. We all know how unpleasant a task it is to be obliged to look over file after file of journals in order to find the information we desire.

This is a work which should be upon the table of every teacher of materia medica and of pharmacy. We bespeak for it a cordial welcome on the part of the medical profession. J. A. M.

